# Acquired Subglottic Cyst in a Premature Infant Following Recurrent Intubation: A Case Report

**DOI:** 10.7759/cureus.105935

**Published:** 2026-03-26

**Authors:** Shahad M Al-Mashjari, Sara M Mashal, Roda Rashid Mohamed Bin S Alshamsi, Naama Waheedi, Shaikha Al Neyadi, Sufian Ghdifan

**Affiliations:** 1 General Practice, Al Qassimi Hospital, Emirates Health Services, Sharjah, ARE; 2 Medical Education, Sheikh Khalifa Medical City, Abu Dhabi, ARE; 3 Graduate Medical Education, Mohammed Bin Rashid University, Dubai, ARE; 4 Pediatric Otolaryngology, Sheikh Khalifa Medical City, Abu Dhabi, ARE; 5 Pediatric Critical Care, Sheikh Khalifa Medical City, Abu Dhabi, ARE; 6 Pediatric Pulmonology, American Hospital Dubai, Dubai, ARE

**Keywords:** airway obstruction, neonatal airway pathology, pediatric otolaryngology, prematurity, recurrent intubation, subglottic cyst

## Abstract

Acquired subglottic cysts are retention cysts that develop as a consequence of iatrogenic trauma to the subglottic mucosa. They are most commonly observed in neonates with a history of prematurity and repeated endotracheal intubation. These cysts may lead to nonspecific symptoms of airway obstruction, such as persistent stridor. We present the case of a premature infant born at 26 weeks of gestation who developed an acquired subglottic cyst following multiple episodes of intubation for recurrent respiratory distress. After persistent respiratory symptoms and an unsuccessful attempt at bronchoscopy, further evaluation using direct laryngoscopy confirmed the presence of a subglottic cyst. This case highlights the importance of recognizing this uncommon but clinically significant condition in premature infants and emphasizes the need for a multidisciplinary approach in diagnosis and management to prevent potentially serious airway complications. It also explores the association between prematurity, low birth weight, and prolonged airway support in the development of subglottic cysts, highlighting the need for further research in this area.

## Introduction

Acquired subglottic cysts are rare but clinically significant lesions that can develop in neonates, particularly premature infants with recurrent intubation and respiratory distress [1]. These cysts, often associated with prolonged mechanical ventilation, present a unique challenge in the management of critically ill infants, as they can lead to life-threatening airway obstruction and exacerbate underlying bronchopulmonary conditions [2]. The pathogenesis of subglottic cysts is thought to be linked to trauma and inflammation of the delicate subglottic mucosa, which is particularly vulnerable to injury during intubation and repeated airway interventions, leading to obstruction of submucosal gland ducts and subsequent cyst formation [3].

Despite advances in neonatal care, the diagnosis and management of these cysts remain complex, requiring a high index of suspicion and a coordinated multidisciplinary approach involving neonatologists, pediatric otolaryngologists, and respiratory specialists [3]. Although relatively uncommon, acquired subglottic cysts have been increasingly recognized in premature infants requiring prolonged airway support. They should be distinguished from other causes of upper airway obstruction, such as laryngomalacia (typically presenting with inspiratory stridor and supraglottic collapse), subglottic stenosis (characterized by fixed airway narrowing), and airway hemangiomas (vascular lesions often associated with progressive biphasic stridor), which may present with similar clinical features but differ in underlying pathology and management.

This case report describes a premature infant with recurrent intubation and mild chronic lung disease of prematurity who developed an acquired subglottic cyst, highlighting the clinical presentation, diagnostic challenges, and therapeutic strategies in this vulnerable population.

## Case presentation

A male infant presented at three months of corrected age with symptoms of acute bronchiolitis. He was born extremely premature at 26+1 weeks of gestation via emergency cesarean section due to placental abruption, with a birth weight of 600 g. His Apgar scores were 5 and 8 at one and five minutes, respectively [4]. The infant’s past medical history was significant for respiratory distress syndrome, bronchopulmonary dysplasia, grade II-III intraventricular hemorrhage, neonatal jaundice, and osteopenia of prematurity. He also had bilateral inguinal hernias and a nonrestrictive penile amniotic band, both of which were surgically repaired. Maternal history was notable for primigravida status and the administration of antenatal dexamethasone. The patient had a history of multiple hospital admissions at different healthcare facilities for recurrent wheezy respiratory illnesses.

On the current presentation, the patient was brought to the emergency department by his parents with a two-day history of cough and runny nose. The cough was dry and persistent, with no associated post-tussive vomiting. He had also developed dyspnea over the preceding day. On examination, he was afebrile, with a heart rate of 120 beats per minute and a respiratory rate of 40-50 breaths per minute. His oxygen saturation was 97% on room air. The infant did not appear pale or cyanosed but exhibited substernal and intercostal retractions. A biphasic stridor was noted. Auscultation revealed bilateral air entry with rhonchi and significant wheezing.

**Figure 1:**
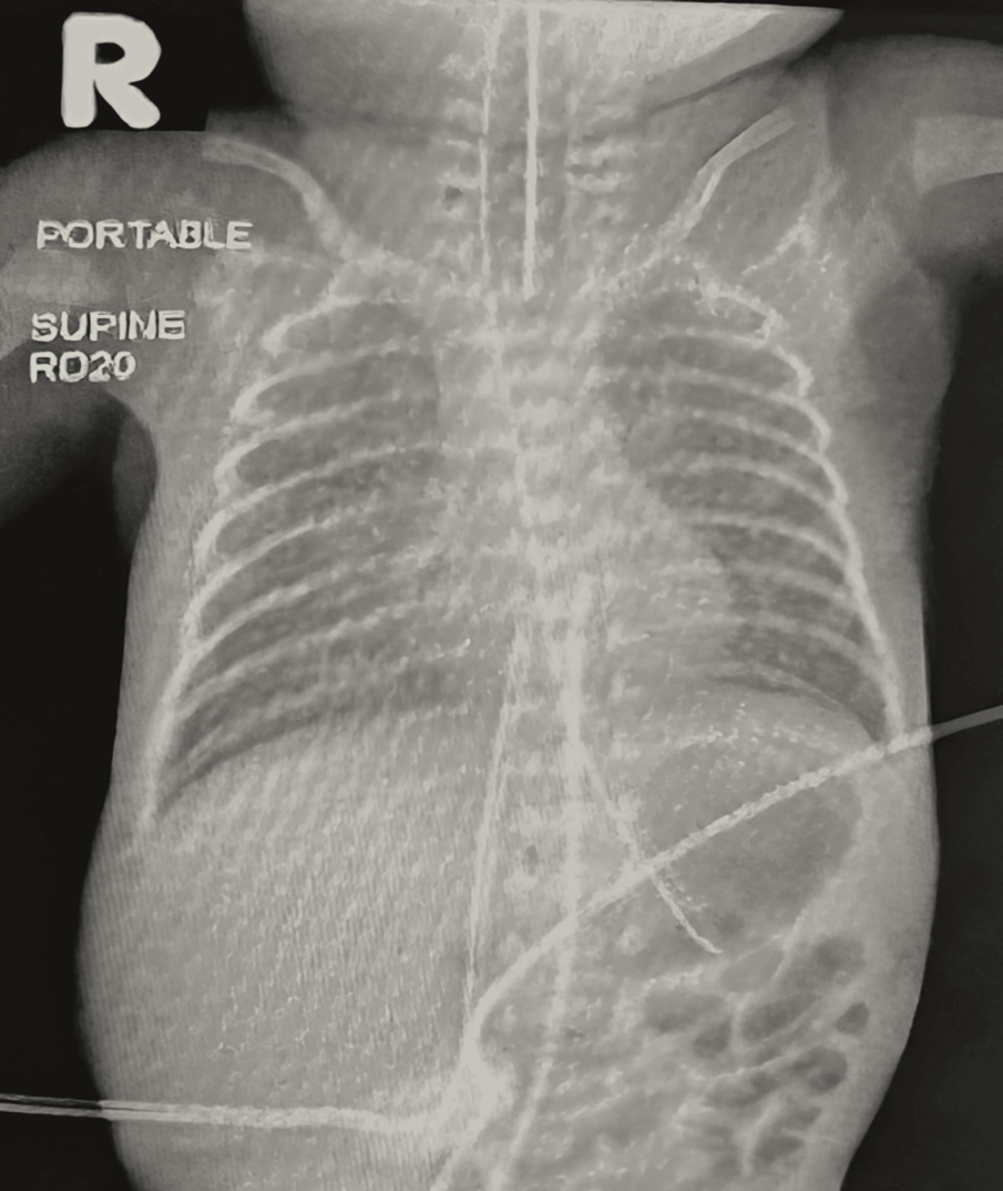
Portable supine chest radiograph of the patient on admission

The patient was admitted to the pediatric ward with a working diagnosis of acute bronchiolitis, with differentials including tracheomalacia or laryngomalacia. He was started on nebulized therapy and analgesia, along with high-flow nasal cannula oxygen and maintenance intravenous fluids. Laboratory investigations performed on admission, including complete blood count, arterial blood gas analysis, and respiratory viral panel, are summarized in Table 1, Table 2, and Table 3. The respiratory viral panel was positive for parainfluenza virus type 1, while other pathogens were not detected. Arterial blood gas analysis demonstrated alkalemia with elevated bicarbonate and mildly elevated pCO₂, suggestive of a primary metabolic process. Chest radiography was unremarkable, with no evidence of consolidation (Figure 1).

**Table 1:** Complete blood count on admission

Parameter	Result	Units	Reference range
White blood cells	7.7	× 10⁹/L	6.0-17.5
Red blood cells	5.04	× 10¹²/L	3.10-4.50
Hemoglobin	120	g/L	105-135
Hematocrit	0.36	L/L	0.33-0.39
Mean corpuscular volume	71.4	fL	70-86
Mean corpuscular hemoglobin	23.8	pg	24-30
Platelet count	374	× 10⁹/L	150-450
Neutrophils (absolute)	5.2	× 10⁹/L	1.0-8.5
Lymphocytes (absolute)	2.32	× 10⁹/L	4.0-13.5

**Table 2:** Arterial blood gas analysis on admission

Parameter	Result	Units	Reference range
pH	7.47	-	7.35-7.45
pCO₂	48.1	mmHg	35-45
pO₂	63.5	mmHg	80-100
O₂ saturation	92.6	%	52-90
HCO₃⁻	33.3	mmol/L	22-26
Base excess	11.2	mmol/L	-2 to +2
Lactate	2.6	mmol/L	0.5-2.0
Glucose	5.2	mmol/L	3.33-5.60
Sodium (Na⁺)	140	mmol/L	134-143
Potassium (K⁺)	5.7	mmol/L	3.4-5.0

**Table 3:** Respiratory viral panel

Pathogen	Result
Adenovirus	Not detected
Coronavirus (229E, HKU1, NL63, and OC43)	Not detected
MERS-CoV	Not detected
SARS-CoV-2	Not detected
Human metapneumovirus	Not detected
Human rhinovirus/enterovirus	Not detected
Influenza A	Not detected
Influenza B	Not detected
Parainfluenza virus 1	Detected
Parainfluenza virus 2	Not detected
Parainfluenza virus 3	Not detected
Parainfluenza virus 4	Not detected
Respiratory syncytial virus	Not detected
Bordetella parapertussis	Not detected
*Bordetella pertussis*	Not detected
*Chlamydia pneumoniae*	Not detected
*Mycoplasma pneumoniae*	Not detected

Over the following days, the patient’s arterial blood gases improved; however, his respiratory symptoms persisted. Examination continued to demonstrate respiratory distress with tachypnea and biphasic stridor. He was subsequently switched to nasal continuous positive airway pressure and intravenous glucocorticoids.

**Figure 2:**
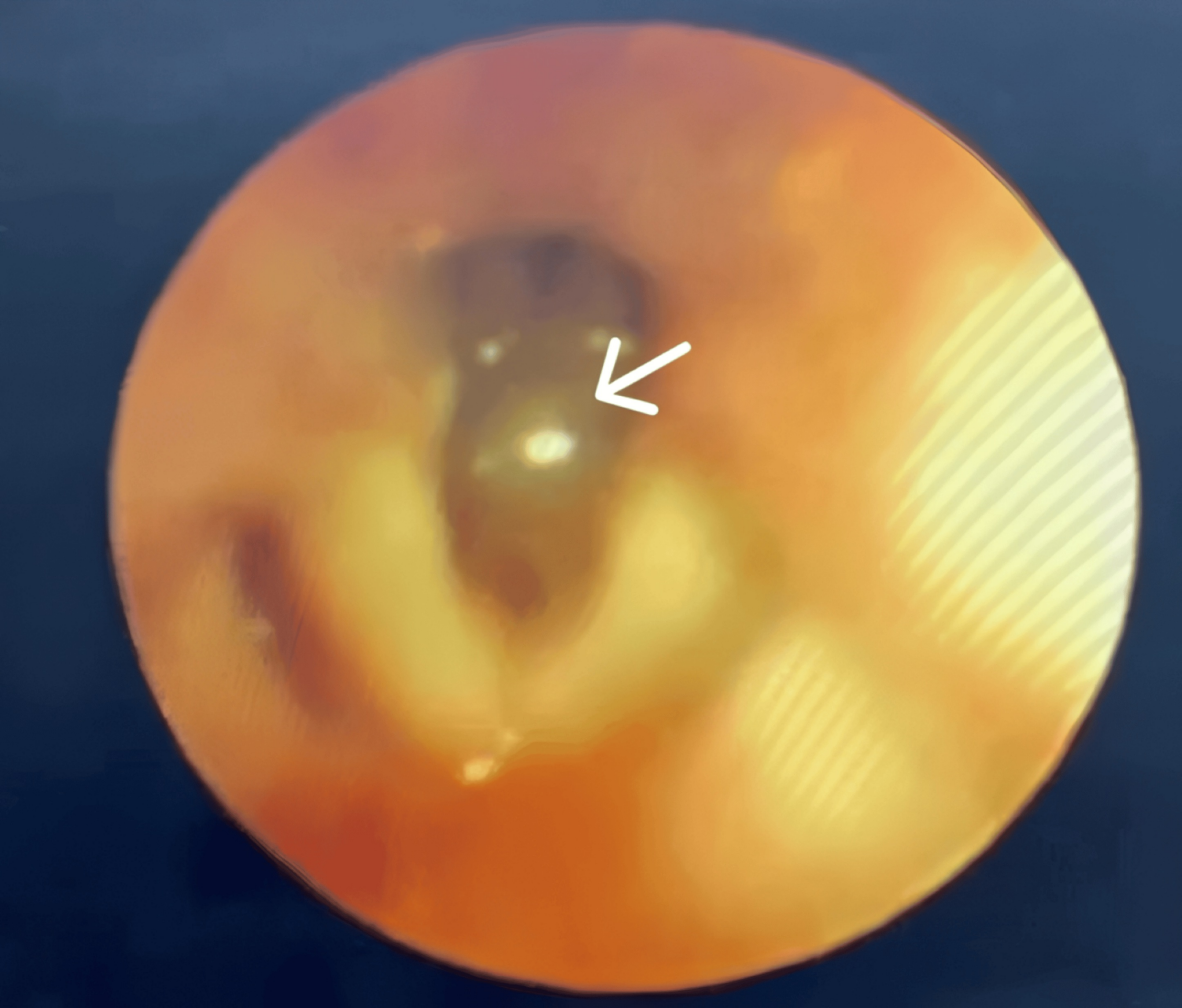
Flexible video laryngoscopy image showing subglottic narrowing and mucosal lesion (white arrow), with normal vocal cords

Due to the lack of clinical improvement, a pediatric pulmonologist was consulted. High-resolution CT of the chest demonstrated consolidation in the right lower lobe and patchy ground-glass opacities in the left lower lobe. A full airway bronchoscopy was attempted but could not be completed due to severe subglottic narrowing. Flexible video laryngoscopy was therefore performed under general anesthesia using a fiber-optic scope. This revealed subglottic narrowing with a mucosal lesion suggestive of a cyst (Figure 2). Posterior laryngitis was also noted, likely secondary to gastroesophageal reflux disease, while the vocal cords appeared normal.

**Figure 3:**
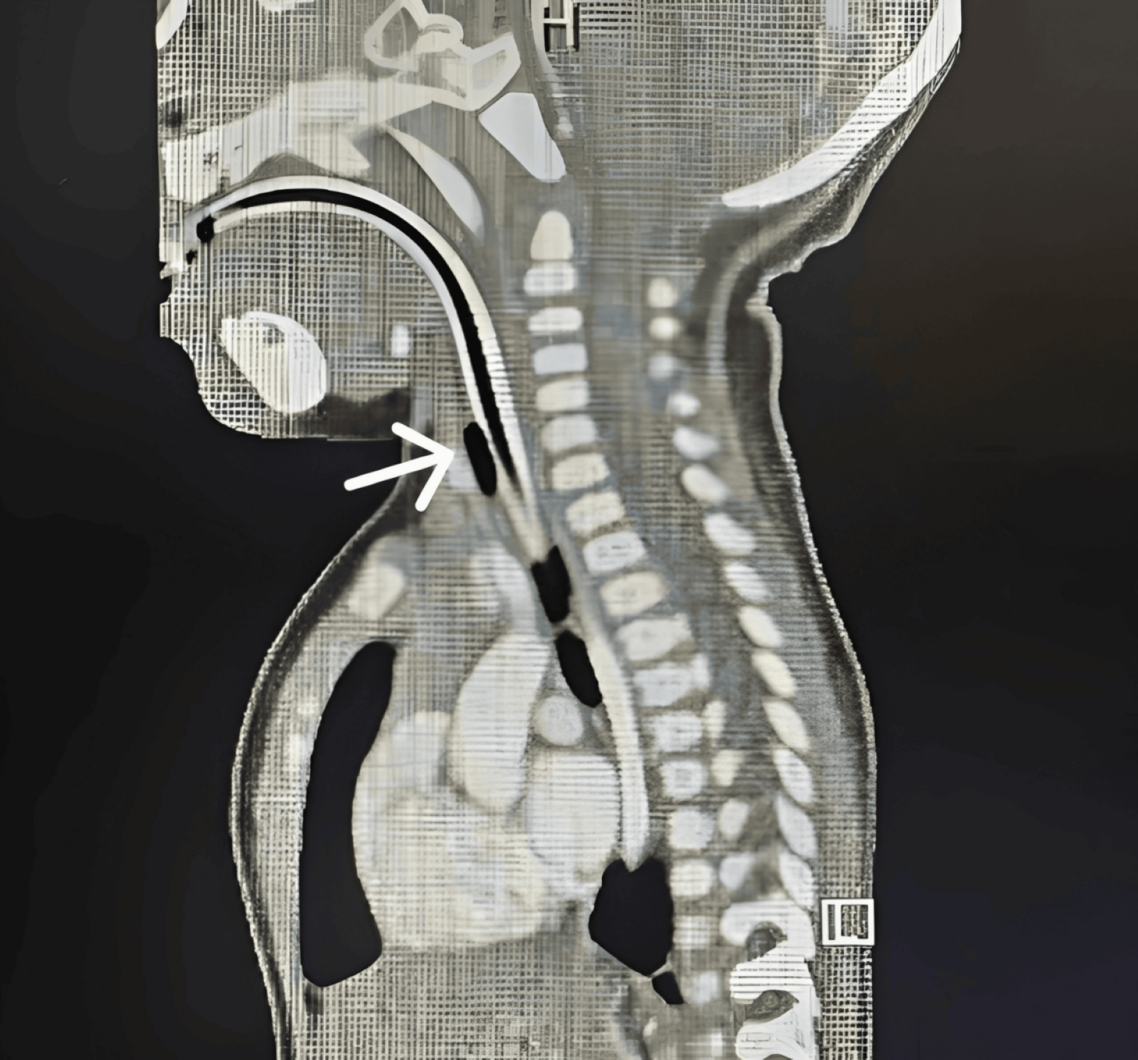
CT of the head and neck showing the upper airway and trachea stented with an endotracheal tube The glottic and subglottic regions are tightly encircling the tube, suggestive of subglottic obstruction; a lesion cannot be excluded.

Following confirmation of the diagnosis of a subglottic cyst, the patient was referred to a tertiary hospital with pediatric ENT services for further management. During the preoperative evaluation, direct laryngoscopy demonstrated a soft, compressible lesion causing approximately 90% subglottic stenosis. A CT scan of the neck with and without contrast also suggested subglottic obstruction (Figure 3). The cyst was subsequently treated with endoscopic marsupialization under microlaryngoscopy, and a biopsy specimen was obtained. The patient’s postoperative recovery was uneventful.

## Discussion

Although relatively rare, acquired subglottic cysts carry significant clinical importance because they can contribute to a wide range of respiratory symptoms. They typically develop following endotracheal intubation. While most cases reported in the literature involve recurrent or prolonged intubation, studies have shown that subglottic cysts may also develop after a single episode of intubation regardless of its duration [2]. These lesions are most commonly observed in premature infants due to the fragility of their airways and the increased likelihood of requiring respiratory support. In this report, we describe a premature infant with recurrent hospital admissions for persistent respiratory symptoms. Despite receiving aggressive medical therapy and respiratory support, his symptoms persisted until direct video laryngoscopy confirmed the presence of a subglottic cyst along with severe subglottic stenosis. This clinical course raised suspicion for an underlying structural airway abnormality. His characteristics, including prematurity, low birth weight, and a history of repeated endotracheal intubation, align with the majority of studies that attribute these factors to the development of subglottic cysts.

Subglottic cysts are defined as mucous retention cysts located below the vocal cords and typically arise as a consequence of mechanical trauma to the airway, most commonly following endotracheal intubation. Their pathophysiology is multifactorial and involves both mechanical injury and the resulting inflammatory response of the subglottic mucosa. One proposed mechanism is the “pneumatic piston effect” of the endotracheal tube [5]. During positive pressure ventilation, the tube moves vertically within the airway, producing repetitive irritation of the mucosa. Over time, this irritation can lead to inflammation, obstruction of mucous glands, and eventual formation of mucous retention cysts. Recent studies have also suggested that frequent suctioning during intubation may contribute to the development of subglottic cysts [6].

Subglottic cysts often present with nonspecific respiratory symptoms. They may initially remain asymptomatic but can progressively enlarge and cause airway obstruction. Stridor is the most commonly reported presenting symptom [7]. Other symptoms may include dyspnea, barking cough, hoarseness of voice, and failure to thrive. Due to the nonspecific nature of these symptoms, subglottic cysts may be misdiagnosed as more common conditions such as asthma or croup. Other differential diagnoses include subglottic stenosis, laryngomalacia, hemangiomas, and foreign body obstruction [7]. Therefore, careful history taking, physical examination, and appropriate investigations are essential to establish the correct diagnosis. In premature infants presenting with persistent or recurrent respiratory symptoms, particularly those with a history of endotracheal intubation, subglottic cysts should be considered as part of the differential diagnosis. Similar presentations have been reported in the literature, where premature infants with a history of intubation presented with persistent stridor and were ultimately diagnosed with subglottic cysts.

Airway bronchoscopy remains the gold standard for identifying subglottic cysts. However, severe airway narrowing may limit the ability to safely perform bronchoscopy, in which case video laryngoscopy can be used as an alternative diagnostic tool. These procedures allow direct visualization of the airway and enable accurate assessment of the location, size, and severity of the lesions. In rare cases, airway instrumentation may exacerbate existing trauma and inflammation [8]. Therefore, careful clinical judgment is required when selecting diagnostic procedures to minimize further airway injury.

The mainstay of treatment for subglottic cysts is endoscopic marsupialization of the cyst [5,9]. In patients with gastroesophageal reflux disease, studies have suggested that the use of concurrent mitomycin C may help reduce the risk of recurrence [10].

Persistent biphasic stridor in an infant should raise concern for underlying structural airway pathology, particularly when symptoms fail to improve despite appropriate medical management. A history of prematurity and previous intubation should further increase suspicion for subglottic cysts, given the strong association between these factors and cyst development reported in the literature. Gastroesophageal reflux disease may also contribute to the formation of subglottic lesions, as repeated exposure of the airway mucosa to gastric acid can lead to chronic inflammation and mucosal injury [6]. Early recognition of these risk factors is important to facilitate timely diagnosis and management, thereby preventing recurrent hospital admissions and progressive airway obstruction.

If left undiagnosed, subglottic cysts may enlarge and cause significant airway obstruction [11]. They are also frequently associated with subglottic stenosis, which further increases the risk of severe respiratory compromise. For this reason, a multidisciplinary approach involving pediatric otolaryngologists, pulmonologists, and intensivists is essential to optimize treatment outcomes and minimize complications in this vulnerable population.

## Conclusions

In our case, the possible factors that contributed to the child’s development of a subglottic cyst were male gender, prematurity, low birth weight, a history of recurrent and prolonged intubation during the neonatal period, and gastroesophageal reflux disease. Our patient most likely underwent recurrent suctioning during his prolonged stay in the NICU while intubated, which correlates with recent studies demonstrating a link between frequent suctioning and the development of subglottic cysts. The patient also had several other comorbidities. It is difficult to determine whether these specific conditions contributed to the development of the subglottic cyst; however, our findings may help provide a foundation for further research exploring potential associations.

Advances in neonatal care have significantly improved survival rates in premature infants, and endotracheal intubation remains a frequently performed procedure in pediatric intensive care settings. As a result, the incidence of subglottic cysts encountered in clinical practice may increase. This highlights the importance of considering subglottic cysts as a potential cause of airway compromise and persistent stridor in premature infants.
